# Prosocial Behaviours and Resilience in School Coexistence: Implications of Creative Self-Efficacy and Stress in Adolescents

**DOI:** 10.3390/bs13120988

**Published:** 2023-11-30

**Authors:** Alba González Moreno, María del Mar Molero Jurado

**Affiliations:** Department of Psychology, University of Almería, 04120 Almería, Spain; mmj130@ual.es

**Keywords:** prosocial behaviour, resilience, school violence, creative self-efficacy, stress, adolescents

## Abstract

Adolescence brings with it a number of problems such as school violence. To reduce stress and increase the well-being of students, it is necessary to enhance certain skills such as prosocial behaviours, resilience, and creative self-efficacy. This cross-sectional study investigated the impact of prosocial behaviours and creative self-efficacy on school violence, stress, and resilience in adolescent students. A total of 743 students aged 14–19 years participated. The results revealed positive correlations between the dimensions of prosocial behaviours, creative self-efficacy, and resilience, and negative correlations with perceived stress. Differences were also observed in the relationship between prosocial behaviours, school violence, and stress. Mediation models indicated that creative self-efficacy acted as a mediator between prosocial behaviours and resilience. In addition, stress was found to moderate the relationship between prosocial behaviours and resilience. This study provides evidence on how prosocial behaviours, resilience, and creative self-efficacy act as a positive element in adolescence.

## 1. Introduction

Adolescence is one of the most sensitive stages in an individual’s life, as it is characterised by multiple changes and challenges in moving from childhood to adulthood [1]. Within this period, social support and positive peer and family relationships are essential for a positive social environment that supports young people’s well-being [2]. One of the most relevant problems today is violence experienced by adolescents in schools, due to the serious damage it entails in adulthood [3]. The occurrence of mental health problems in adulthood is related to exposure to violence in childhood and adolescence [4]. Studies indicate that problems such as suicidal ideation caused by exposure to violence can be reduced by increasing the resilience of young people [5]. Another aspect of adolescence that can interfere with positive adolescent development is stress, as young people with high levels of perceived stress are more likely to develop a mental disorder [6,7]. One of the capacities to counteract stress, helping to adapt to setbacks and overcome adversity, is resilience [8]. Young people who have been victims of bullying show a worse state of emotional well-being, with resilience acting as a moderator between such victimisation and well-being [9,10]. This idea is also presented in other study, where it is indicated that being a victim of bullying is a predictor of subsequent mental health problems [11]. Some factors such as empathy, family support, or life satisfaction reduce violent attitudes in adolescence [12], as occurs in other contexts such as healthcare [13]. Thus, given the different problems experienced by adolescents, it is necessary to promote those skills that are beneficial in their daily lives [14,15].

Creativity refers to the ability to generate multiple ideas or solutions to a problem [16]. In line with this idea, authors such as Trefinger et al. [17] interpreted creativity as the consequence of a set of strategies that people use to reason, make decisions, solve problems, or make sense of life, and therefore considered it necessary to examine creativity in relation to productive structures of thought. Osborn [18] also previously proposed this link between creativity and conflict resolution, and proposed his own model called Creative Problem Solving (CPS), which shows how people use six steps to solve a problem: problem finding, identifying the facts, defining the problem, brainstorming, solution finding, and solution implementation. Self-efficacy is recognised as a component of resilience that helps in coping with post-traumatic events [19,20,21]. Thus, the term creative self-efficacy refers to people’s self-judgements about their creative expressions and their abilities to produce useful and novel outcomes [22,23]. Creativity is estimated to predict a positive emotional state, as well as being linked to better academic performance [24]. In addition, creativity has been found to be an element that moderates resilience and, thus, acts positively on self-efficacy in the face of vulnerable moments that have a great impact on mental health, such as the COVID-19 pandemic [25]. Another aspect to highlight, focusing on creativity as a component that improves quality of life, is how this construct fosters positive emotions and reduces perceived stress [26].

In terms of variables that promote well-being in adolescent students, prosocial behaviours are significantly associated with lower levels of depression [27]. Prosocial behaviours refer to those positive attitudes that are voluntarily performed with the intention of helping others and without any benefit in return [28]. The relationship between prosocial behaviours and their efficacy in social relationships is found in previous studies, which indicate that adolescents with higher empathy report higher prosocial behaviour [29]. Studies indicate that there is a positive correlation between all the different dimensions of prosocial behaviour and adolescent students’ perception of school climate [30]. Prosocial behaviours are considered to promote personal well-being in adolescence, as dimensions of prosocial behaviour have been found to be predictors of both subjective and psychological well-being [31].

### Aim and Hypothesis of the Study

This research aims to identify how prosocial behaviours and creative self-efficacy act on school violence, stress, and resilience in adolescent students. The initial hypotheses considered are the following:

**H1:**  *Prosocial behaviours and creative self-efficacy are negatively correlated with perceived stress and positively correlated with resilience.*

**H2:**  *There are differences according to prosocial behaviour and creative self-efficacy in school violence roles (bully, victim, and observer).*

**H3:**  *Creative self-efficacy mediates the dimensions of prosocial behaviour and resilience.*

**H4:**  *Stress moderates the relationship between different dimensions of prosocial behaviours and resilience.*

## 2. Materials and Methods

### 2.1. Study Design and Participants

This quantitative study was conducted using a descriptive cross-sectional design. To test the reliability of the study, the guidelines of the STROBE statement for cross-sectional studies were followed [32]. The study participants, 743 adolescent students aged 14–19 years (*M* = 14.99, *SD* = 0.86), were selected using cluster sampling. In terms of the total sample, 50.7% were girls (*n* = 377) and 49.3% boys (*n* = 366). All participants were enrolled in different public secondary schools in the province of Almería (Spain). In terms of academic year, 50.7% of these students were in the third year and 49.1% were in the fourth year. The most predominant nationality in the sample was Spanish (92.9%), although there were also students of other origins such as Moroccans, Mexicans, and Russians.

### 2.2. Instruments

The different variables included in this research were assessed by means of a dossier containing an ad hoc section with a series of specific questions and the previously validated instruments. The variables assessed in this study were as follows:

- School violence. Students’ perceptions of school violence were investigated through questions included in an ad hoc survey. The questions were answered dichotomously and focused on three roles: victim (e.g., “Have you experienced violence from your peers?”), bully (e.g., “Have you exercised violence to your peers?”), and observer (e.g., “Have you observed violence towards other peers?”).
- Prosocial behaviour. Prosocial behaviour in young people was assessed using the Prosocial Behaviour Questionnaire (PCQ) [33]. Aimed at adolescents aged 10–17 years, the questionnaire measures the participants’ use of certain types of helping, such as sharing, cooperating, understanding, and encouraging. The scale has a total of 55 items and consists of four dimensions answered on a Likert-type scale with four options ranging from ‘never’ to ‘always’. The first dimension, empathy, is a 20-item measure of one’s ability to put oneself in the place of others and alleviate their discomfort (e.g., “I help people who have problems”). The second dimension is respect, which consists of 16 items and refers to the ability to treat others fairly (e.g., “When I am wrong, I admit it”). The third dimension, called social relations, consists of 11 items and focuses on understanding the ability to establish positive social relationships (e.g., “I like to talk to friends and colleagues”). Finally, the fourth dimension is leadership, which refers to the ability to lead and organise team activities (e.g., “When something needs to be done, I initiate it”). This manifesto does not provide overall scores, but individual scores for each category. Internal consistency was acceptable and excellent for the following dimensions: empathy (α = 0.90), respect (α = 0.78), social relations (α = 0.69), and leadership (α = 0.74).
- Creative self-efficacy. Creative self-efficacy was measured using the Creative Self-Efficacy Scale [34]. This scale has five items (e.g., “I am confident that I can generate original and appropriate ideas”) which are answered on a four-point Likert scale. The total score obtained corresponds to the perception of creative self-efficacy. The instrument achieved a reliability level of *α* = 0.64.
- Stress. The level of stress perceived by the participants was assessed using the Spanish adaptation of the Student Stress Inventory Scale (SSI-SM) [35] developed by Escobar et al. [36]. This instrument consists of a total of 22 items that are answered using a five-point Likert scale (1 = not at all; 2 = rarely; 3 = sometimes; 4 = often; 5 = completely). The stress manifestations scale covers both a total stress score and a three-factor score: emotional manifestations (e.g., “I feel irritated”), physiological manifestations (e.g., “I lose my voice or become hoarse”), and behavioural manifestations (e.g., “I act defensive towards others”). A good internal consistency was obtained for the total scale (*α* = 0.89) and the emotional manifestations dimension (*α* = 0.87), acceptable for physiological manifestations (*α* = 0.71), and questionable for behavioural manifestations (*α* = 0.65).
- Resilience. The resilience of young people was assessed using the reduced variant CD-RISC10 [37] which measures resilience in a global way that has been developed from the original Connor–Davidson scale (CD-RISC) [38]. This scale measures the human capacity to cope with traumatic situations (e.g., “I am able to adapt when changes arise”), using ten items that are answered on a Likert scale with four response options. Internal consistency was good (*α* = 0.83).

### 2.3. Procedure and Data Collection

Once the instruments had been selected and the data collection booklets had been prepared, we contacted several secondary schools in different municipalities in the province of Almería (Spain). Six of the schools agreed to participate in the study. A meeting date was arranged with the management of each centre so that the students themselves could complete the data collection booklets.

Data collection was carried out in person and individually by the students in their respective schools. All students and their legal guardians were previously informed about the purpose of the study and provided their consent before data collection began. It should be noted that of the initial 806 students who participated in the data collection process, 743 were finally included in this study. The remaining ones were excluded due to the lack of information required for the analysis.

The anonymity of the students was guaranteed, since at no time did the participants have to reveal any personal data. All data obtained were collected in a general database. Data collection took place during a period that spanned from February to June 2022. In addition, it is relevant to mention that this study received the approval of the Bioethics Committee on Human Research of the University of Almería, with reference UALBIO2021/025, thus guaranteeing ethical standards in research.

### 2.4. Data Analysis

Data analysis was performed using SPSS version 28 statistical software [39]. Cronbach’s alpha coefficient was used to test the reliability of the tools used. This coefficient is interpreted as follows: <0.5 unacceptable, >0.5 poor, >0.6 questionable, >0.7 acceptable, >0.8 good, and >0.9 excellent [40]. Descriptive analysis was conducted to provide relevant information about the students who participated in this study. In addition, Pearson’s bivariate correlation analysis was performed to determine whether there was a relationship between the variables studied. The absolute values obtained are interpreted in the following categories: no correlation between 0 and 0.10; weak correlation between 0.10 and 0.29; moderate correlation between 0.30 and 0.50; and strong final correlation between 0.50 and 1.00 [41].

In addition, Welch’s *t*-test used to compare samples with different variances was performed. This test was conducted to examine differences between the different school violence roles (bully, victim, and observer) and the variables analysed (prosocial behaviour, creative self-efficacy, stress, and resilience). Cohen’s *d* was calculated to estimate effect sizes: small 0.50, small 0.50, moderate 0.50–0.80, and large ≥ 0.80 [42].

Subsequently, in order to identify the behaviour of creative self-efficacy as a mediating variable and stress as a moderating variable in the relationship established between prosocial behaviours (empathy, respect, social relations, and leadership) and resilience, a series of mediation and moderation analyses were carried out, respectively. For this purpose, the medmod module integrated in jamovi v.2.3.2 [43] was used, which allows the computation of models on mediation and moderation effects, providing information on standardised coefficients, z-scores, and significance levels. In both cases, the bootstrapping technique was applied with estimated coefficients from 5000 bootstrap samples, with a 95% confidence interval.

## 3. Results

### 3.1. Descriptive Analyses and Correlations

Table 1 shows the correlations between the different variables analysed. These results show how the dimensions of prosocial behaviour (empathy, respect, social relations, and leadership) correlate positively with creative self-efficacy and resilience. Regarding the association between prosocial behaviours and stress, a negative relationship was obtained between the dimensions of respect and social relations with all dimensions of stressors (emotional manifestations, physiological manifestations, and behavioural manifestations) and total stress. On the other hand, empathy correlates positively with emotional and physiological manifestations, but negatively with behavioural manifestations. There is no correlation between empathy and overall stress score. Finally, leadership was negatively correlated with emotional and physiological manifestations and total stress; no such correlation was obtained with behavioural manifestations.

Regarding creative self-efficacy, the results indicate a significant positive relationship with all dimensions of prosocial behaviour and with resilience. On the other hand, a significant negative correlation was obtained with the emotional manifestations dimension and the total stress score.

With regard to stress, apart from the above, its negative relationship with resilience is highlighted. Thus, resilience is correlated with all the variables examined: positively with prosocial behaviour and creativity, and negatively with stress.

### 3.2. Differences between School Violence Roles and the Examined Variables

Before diving into the differences found in the different roles of school violence, it is crucial to highlight an important fact. In our sample, the number of adolescents who admitted to having carried out violent behaviours with their peers is considerably lower (*n* = 57) compared to those who claimed not to have executed this type of violent behaviour (*n* = 676). The same happens in the case of the victim role, as we obtained a more limited number of adolescents who acknowledged being victims (*n* = 97) compared to those who stated that they had not been bullied (*n* = 635). However, these differences are not as marked in the case of the observer role. Here, the number of adolescents who have observed situations of school violence (*n* = 332) does not differ significantly from those who have not witnessed such situations (*n* = 398). In view of these differences, Welch’s *t*-test was performed to determine which roles of violence were more significant among the variables included in the study. In addition, this information was completed by adding the differences according to sex to the different subgroups: aggressive students, victim students, and observer students.

The data in Table 2 show that adolescents who do not bully their peers have a higher level of empathy and respect. Another detail to note is how those young people who are not victims have higher levels of social relations than those who are victims of school violence. On the other hand, observers are more empathetic than non-observers who show greater respect. It is noteworthy how there are no significant differences in the leadership dimension between the roles of violence.

Considering the differences according to sex, it was found that female victims scored higher in empathy (*M* = 60.61; *SD* = 8.53) and respect (*M* = 50.11; *SD* = 6.69) than male victims (empathy: *M* = 53.84; *SD* = 10.31/respect: *M* = 45.83; *SD* = 7.12). The same is true for girl observers of violence, who also score higher in empathy (*M* = 59.27; *SD* = 9.33) and respect (*M* = 49.14; *SD* = 6.30) than boy observers (empathy: *M* = 54.46; *SD* = 9.33/respect: *M* = 47.26; *SD* = 7.24).

The differences obtained between the level of creative self-efficacy and the different roles of violence can be seen in Table 3. These results indicate that there are no statistically significant differences between the level of creative self-efficacy and the different violence roles examined. Such differences have not been found in the different subgroups of aggressors, victims, and observers in relation to sex either.

The results obtained between stress and roles of violence are shown in Table 4. Adolescents who are both victims and observers of violence have higher levels of stress in all its dimensions (emotional manifestations, physiological manifestations, and behavioural manifestations) and in stress. In addition, it was found that bullies scored higher in behavioural manifestations than non-bullies. However, there are no significant differences in the dimensions of emotional and physiological manifestations and total stress between bullies and non-bullies.

Considering the differences according to sex in the different roles of school violence, it was found that there are no differences by sex in the aggressor students in the stress variable. On the other hand, girl victims show higher emotional manifestations (*M* = 35.56; *SD* = 7.31) compared to boy victims (*M* = 32.28; *SD* = 8.64). However, in behavioural manifestations it is the boy victims who present higher scores (*M* = 14.50; *SD* = 4.50) than the girl victims (*M* = 12.23; *SD* = 3.17). Regarding the role of observer, differences were also found according to sex. Observer girls scored higher in emotional manifestations (*M* = 34.07; *SD* = 7.96), physiological manifestations (*M* = 14.81; *SD* = 4.49), and total stress (*M* = 61.18; *SD* = 13.77) than boy observer (emotional manifestations: *M* = 30.29; *SD* = 8.42; physiological manifestations: *M* = 13.83; *SD* = 4.79; total stress: *M* = 57.46; *SD* = 15.30).

After analysing the data on the differences between resilience and the roles of violence (Table 5), no significant differences were found for any of the roles in relation to this variable. As for differences according to sex and roles of violence, no significant differences were found either.

### 3.3. The Mediating Role of Creative Self-Efficacy in the Relationship between Prosocial Behaviour and Resilience

**Figure 1:**
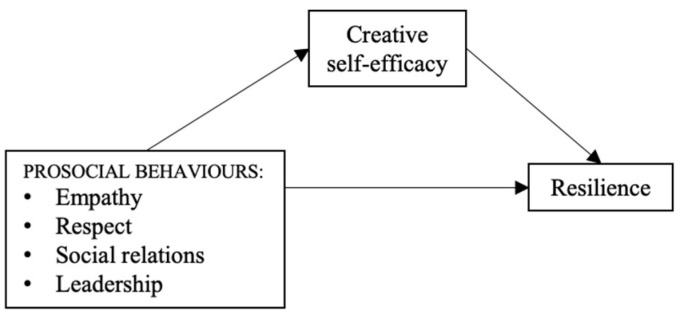
Hypothetical model of the mediation of creative self-efficacy in the relationship between prosocial behaviours and resilience.

Based on the results obtained, we set out to assess whether creative self-efficacy may be mediating the relationship established between prosocial behaviours and resilience in adolescents. To do so, we computed different simple mediation models, taking as independent variables, in each case, the prosocial behaviours: empathy (*X*_1_), respect (*X*_2_), social relations (*X*_3_), and leadership (*X*_4_). Common to all models, creative self-efficacy is presented as a mediator (*M*). Finally, resilience is presented as the dependent variable (*Y*) (Figure 1).

**Figure 2:**
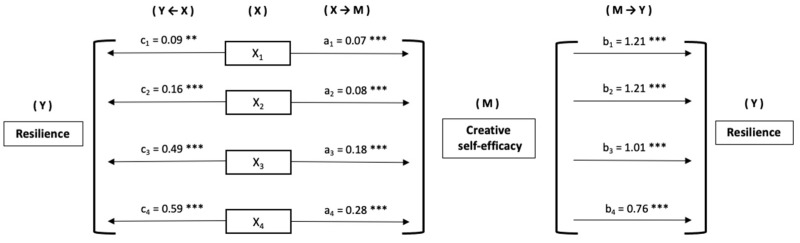
Mediation models and path estimates [Note. *X*_1_ = Empathy; *X*_2_ = Respect; *X*_3_ = Social relations; *X*_4_ = Leadership. ** *p* < 0.01; *** *p* < 0.001].

The estimation of direct effects (*X* → *Y*) reveals the existence of significant relationships of all prosocial behaviours with resilience (Figure 2). As for indirect effects (*X* → M → *Y*), significance is obtained for all prosocial behaviours as follows: empathy *β* = 0.09, *SE* = 0.013, 95% *CI* (0.065, 0.119), *Z* = 6.71, *p* < 0.001, 49.8%_Md_; respect *β* = 0.10, *SE* = 0.019, 95% *CI* (0.066, 0.141), *Z* = 5.42, *p* < 0.001, 39.3%_Md_; social relations *β* = 0.18, *SE* = 0.027, 95% *CI* (0.129, 0.235), *Z* = 6.66, *p* < 0.001, 26.9%_Md_; and leadership *β* = 0.21, *SE* = 0.032, 95% *CI* (0.154, 0.281), *Z* = 6.59, *p* < 0.001, 26.7%_Md_.

### 3.4. The Moderating Effect of Stress on the Predictive Value of Prosocial Behaviours on Resilience

**Figure 3:**
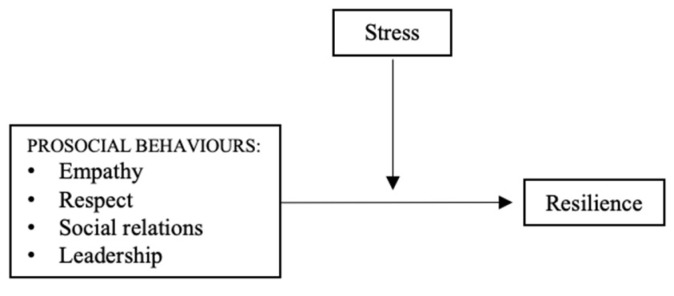
Hypothetical model of stress moderation in the relationship between prosocial behaviours and resilience.

Based on the simple moderation models, the coefficients of the effects of each of the independent variables (empathy, respect, social relations, and leadership), of the moderating variable (stress), and of the interaction term on the dependent variable (resilience) are estimated in each case. Figure 3 graphically presents the proposed theoretical model, with the variables involved in the simple moderation analyses.

In the first analysis, prosocial behaviours were included as a predictor variable in each case, and resilience as a criterion variable, considering the moderating role of stress. In all cases, prosocial behaviours establish a positive and significant relationship with resilience: empathy (*β* = 0.19; *SE* = 0.026; *z* = 7.22; *p* < 0.001), respect (*β* = 0.18; *SE* = 0.039; *z* = 4.60; *p* < 0.001), social relationships (*β* = 0.56; *SE* = 0.053; *z* = 10.58; *p* < 0.001), and leadership (*β* = 0.74; *SE* = 0.048; *z* = 15.29; *p* < 0.001).

In addition, the results reported a moderating effect of stress on the relationship between respect and resilience: respect * stress (*β* = −0.008; *SE* = 0.002; *z* = −3.35; *p* < 0.001). Specifically, the effect of respect as a predictor on the dependent variable (resilience) was observed to be different for different levels of stress as a moderator, being significant at medium (*β* = 0.18; *SE* = 0.039; *z* = 4.57; *p* < 0.001) and high levels of stress (*β* = 0.30; *SE* = 0.047; *z* = 6.46; *p* < 0.001). For the rest of the prosocial behaviours, the moderating effect of stress on resilience was not verified. Therefore, no significant data were obtained with empathy, social relations, or leadership.

## 4. Discussion and Conclusions

This research has allowed us to verify the role that variables such as creative self-efficacy and prosocial behaviours in adolescence have on school violence, stress, and resilience in adolescent students. We wanted to investigate these variables because adolescence is a sensitive stage in which it is necessary to promote those skills that are beneficial for young people [1,14,15]. To this end, four initial hypotheses were proposed and subsequently analysed.

The first hypothesis was that prosocial behaviours and creative self-efficacy are negatively correlated with stress and positively correlated with resilience. This hypothesis, when tested, was accepted. Two dimensions of prosocial behaviour (respect and social relations) were found to be negatively related to the different dimensions of stressors and total stress. Furthermore, all dimensions of prosocial behaviour were positively related to resilience. These ideas can be linked to previous studies indicating that prosocial behaviours promote personal well-being in adolescence [31]. On the other hand, the first hypothesis was also accepted in relation to creative self-efficacy. The results indicate that creative self-efficacy is negatively related to the dimension of emotional manifestations and total stress, and positively related to resilience. This idea is linked to the fact that creativity is a problem-solving ability and predicts a positive emotional state [16,17,18,24].

The second hypothesis of this study was focused on finding out the differences according to the role of violence used (bully, victim, and observer) in each of the variables analysed. This initial hypothesis established that there are differences according to these roles in prosocial behaviour and creative self-efficacy. Regarding prosocial behaviour, it was found that non-bullies and observers have more empathy; non-bullies and non-observers have more respect; and non-victims have a higher level of social relations. As can be seen, those who do not directly participate in school violence have higher levels of prosocial behaviour. This is a positive finding, as previous studies indicate that exposure to violence results in mental health problems [4]. In addition, a high level of prosocial behaviour is related to lower depression, and a positive environment supports young people’s well-being [2,27]. On the other hand, in relation to creative self-efficacy, this hypothesis was not accepted, because no significant differences with school violence roles were obtained. Creative self-efficacy is a complex psychological characteristic that may vary widely among individuals, regardless of their role in school violence situations. Individual variability may be greater than differences related to violence roles, making it difficult to detect statistically significant differences. One aspect to highlight is that it is the girls who are victims and observers who have higher scores in the different dimensions of stress and prosocial behaviours compared to the male sex. This may be due to gender expectations that encourage emotional expression and empathy in girls, as well as socialization that encourages them to care for and help others.

With regard to hypothesis three, this was focused on the fact that creative self-efficacy acts as a mediator of prosocial behaviour and resilience. The results obtained support this hypothesis, as the relationship between all dimensions of prosocial behaviour and resilience is mediated by creative self-efficacy. This idea is linked to previous studies that indicate that creativity is a positive element in resilience, thus acting effectively in times of vulnerability [25]. Moreover, creativity is estimated to promote positive emotions and reduce perceived stress [26].

Finally, hypothesis four referred to stress moderating the different dimensions of prosocial behaviours and resilience. The results show that there is such moderation of stress in its relation to resilience only with the dimension of respect. It is estimated that a high level of stress can develop a mental disorder [6], as well as that increasing resilience in young people helps to reduce suicidal ideation in young people [5]. The reason why stress moderates the relationship between resilience and the respect dimension of prosocial behaviours, and not necessarily other dimensions such as empathy, social relationships, and leadership, may be multifactorial and require further analysis to fully understand its underlying complexities. It is possible that the respect dimension is particularly sensitive to the effects of stress due to its specific characteristics. For example, respect may be more closely related to perceived control over stressful situations or to resilience in the face of stress.

In conclusion, it is worth highlighting how this research has allowed us to investigate variables such as prosocial behaviours and creative self-efficacy in adolescence. One of the practical implications of this work could be to know those variables that are beneficial in aspects such as the well-being or mental health of adolescents. Taking into account the limitations obtained, it is necessary to point out the scarcity of previous studies on creative self-efficacy in the educational setting. However, it is important to point out, as part of the limitations of the study, that the same person can assume different roles in school violence, including being a victim, aggressor, and observer, or any combination of these three roles. This complexity in role interactions may affect how we interpret the results and understand how these variables are related in adolescence. In addition, another significant limitation is that the Creative Self-Efficacy Scale used in this study showed moderate reliability. This means that, although this measure can provide valuable information about adolescents’ creative self-efficacy, it is not entirely accurate and there may be some margin of error in the scores obtained. In future research, it would be advisable to explore the use of alternative or complementary measures to assess creative self-efficacy more accurately. In addition, these future lines of research could focus on delving deeper into school violence role interactions, exploring the influence of external factors on the variables studied, such as prosocial behaviours and creative self-efficacy, and conducting long-term follow-up studies to assess the lifelong impact of these factors on youth development. Such research could provide valuable insights for improving adolescent mental health and well-being, as well as for designing effective interventions in educational settings.

## Figures and Tables

**Table 1:** Descriptives and correlation matrix between prosocial behaviour, creativity, stress, and resilience (*N* = 743).

		[1]	[2]	[3]	[4]	[5]	[6]	[7]	[8]	[9]	[10]
Prosocial Behaviour	[1] Empathy	-									
	[2] Respect	0.53 ***	-								
	[3] Social Relations	0.39 ***	0.28 ***	-							
	[4] Leadership	0.35 ***	0.16 ***	0.53 ***	-						
Creative Self-Efficacy	[5] Total Auto. Creative	0.29 ***	0.22 ***	0.33 ***	0.51 ***	-					
Stress	[6] Emotional Manifestations	0.09 *	−0.16 ***	−0.35 ***	−0.24 ***	−0.12 ***	-				
	[7] Physiological Manifestations	0.08 *	−0.15 ***	−0.24 ***	−0.09 *	−0.05	0.69 ***	-			
	[8] Behavioural Manifestations	−0.16 ***	−0.43 ***	−0.20 ***	−0.04	−0.00	0.51 ***	0.54 ***	-		
	[9] Total Stress	0.04	−0.25 ***	−0.33 ***	−0.18 ***	−0.09 *	0.93 ***	0.85 ***	0.72 ***	-	
Resilience	[10] Total Resilience	0.25 ***	0.24 ***	0.44 ***	0.52 ***	0.46 ***	−0.36 ***	−0.23 ***	−0.14 ***	−0.32 ***	-
	Mean	55.98	48.91	32.78	20.57	17.65	30.88	13.50	12.18	56.55	26.65
	*SD*	9.62	6.62	4.66	4.52	4.27	8.61	4.54	3.71	14.65	7.00
	Min.	21	27	19	8	1	11	6	6	23	5
	Max.	75	77	44	32	29	55	29	30	103	40

*SD* = Standard Deviation; Min = Minimum; Max = Maximum; *** *p* < 0.001; * *p* < 0.05.

**Table 2:** Differences between prosocial behaviours and violence roles.

Roles of Violence			Prosocial Behaviours			
			Empathy	Respect	Social Relations	Leadership
Bully	Harasses	Mean	53.13	44.13	31.98	21.07
		*SD*	10.74	7.54	4.94	4.76
	Does not harass	Mean	56,22	49.33	32.84	20.53
		*SD*	9.41	6.39	4.65	4.52
	Welch		2.10 *	5.04 ***	1.26	−0.82
	*p*		0.040	<0.001	0.211	0.411
	*d*		0.30	0.80	-	-
Victim	Is a victim	Mean	57.12	47.91	31.63	20.48
		*SD*	10.04	7.21	4.95	4.90
	Not a victim	Mean	55.90	49.13	32.96	20.60
		*SD*	9.45	6.49	4.61	4.50
	Welch		−1.12	1.57	2.49 **	0.24
	*p*		0.263	0.118	0.014	0.808
	*d*		-	-	0.27	-
Observer	Has observed	Mean	57.04	48.27	32.74	20.86
		*SD*	9.62	6.81	4.45	4.60
	Has not observed	Mean	55.29	49.60	32.82	20.32
		*SD*	9.26	6.40	4.84	4.47
	Welch		−2.49 *	2.68 **	0.23	−1.59
	*p*		0.013	0.007	0.812	0.112
	*d*		−0.18	0.20	-	-

*SD* = Standard Deviation *** *p* < 0.001; ** *p* < 0.01; * *p* < 0.05.

**Table 3:** Differences between creative self-efficacy and roles of violence.

Roles of Violence			Creative Self-Efficacy
Bully	Harasses	Mean	15.02
		*SD*	2.60
	Does not harass	Mean	14.75
		*SD*	2.50
	Welch		−0.77
	*p*		0.443
	*d*		-
Victim	Is a victim	Mean	15.13
		*SD*	2.58
	Not a victim	Mean	14.73
		*SD*	2.49
	Welch		−1.42
	*p*		0.157
	*d*		-
Observer	Has observed	Mean	14.88
		*SD*	2.42
	Has not observed	Mean	14.72
		*SD*	2.58
	Welch		−0.86
	*p*		0.389
	*d*		-

*SD* = Standard Deviation.

**Table 4:** Differences between stress and the roles of violence.

Roles of Violence			Stress			
			Emotional Manifestations	Physiological Manifestations	Behavioural Manifestations	Total Stress
Bully	Harasses	Mean	31.71	14.00	14.44	60.15
		*SD*	6.97	4.84	4.29	13.32
	Does not harass	Mean	30.78	13.44	11.96	56.18
		*SD*	8.76	4.50	3.60	14.75
	Welch		−0.94	−0.84	−4.23 ***	−2.14
	*p*		0.346	0.401	<0.001	0.036
	*d*		-	-	0.58	-
Victim	Is a victim	Mean	33.87	15.44	13.40	62.71
		*SD*	8.15	4.63	4.06	14.14
	Not a victim	Mean	30.42	13.18	11.95	55.55
		*SD*	8.64	4.47	3.62	14.59
	Welch		−3.85 ***	−4.47 ***	−3.33 ***	−4.62 ***
	*p*		<0.001	<0.001	0.001	<0.001
	*d*		−0.41	−0.49	−0.37	−0.49
Observer	Has observed	Mean	32.32	14.36	12.78	59.46
		*SD*	8.38	4.65	3.78	14.60
	Has not observed	Mean	29.64	12.70	11.60	53.94
		*SD*	8.60	4.29	3.56	14.22
	Welch		−4.24 ***	−4.94 ***	−4.31 ***	−5.14 ***
	*p*		<0.001	<0.001	<0.001	<0.001
	*d*		−0.31	−0.36	−0.32	−0.38

*SD* = Standard Deviation; *** *p* < 0.001.

**Table 5:** Differences between resilience and roles of violence.

Roles of Violence			Resilience
Bully	Harasses	Mean	26.75
		*SD*	7.74
	Does not harass	Mean	26.62
		*SD*	6.97
	Welch		−0.11
	*p*		0.909
	*d*		-
Victim	Is a victim	Mean	25.65
		*SD*	8.08
	Not a victim	Mean	26.82
		*SD*	6.83
	Welch		1.36
	*p*		0.176
	*d*		-
Observer	Has observed	Mean	26.34
		*SD*	7.31
	Has not observed	Mean	26.96
		*SD*	6.73
	Welch		1.19
	*p*		0.234
	*d*		-

*SD* = Standard Deviation.

## Data Availability

The data presented in this study are available on request from the corresponding author.
